# Policy and programmatic implications of task shifting in Uganda: a case study

**DOI:** 10.1186/1472-6963-12-61

**Published:** 2012-03-12

**Authors:** Yoswa M Dambisya, Sheillah Matinhure

**Affiliations:** 1 Health Systems Research Group, Department of Pharmacy, University of Limpopo, Private Bag X1106, Sovenga 0727, South Africa; 2 East, Central and Southern African Health Community (ECSA HC), Human Resources & Capacity Building Programme, ECSA HC Secretariat, 157 Olorien, Njiro Road, Arusha, Tanzania, East Africa

## Abstract

**Background:**

Uganda has a severe health worker shortage and a high demand for health care services. This study aimed to assess the policy and programmatic implications of task shifting in Uganda.

**Methods:**

This was a qualitative, descriptive study through 34 key informant interviews and eight (8) focus group discussions, with participants from various levels of the health system.

**Results:**

Policy makers understood task shifting, but front-line health workers had misconceptions on the meaning and intention(s) of task shifting. Examples were cited of task shifting within the Ugandan health system, some formalized (e.g. psychiatric clinical officers), and some informal ones (e.g. nurses inserting IV lines and initiating treatment). There was apparently high acceptance of task shifting in HIV/AIDS service delivery, with involvement of community health workers (CHW) and PLWHA in care and support of AIDS patients.

There was no written policy or guidelines on task shifting, but the policy environment was reportedly conducive with plans to develop a policy and guidelines on task shifting.

Factors favouring task shifting included successful examples of task shifting, proper referral channels, the need for services, scarcity of skills and focused initiatives such as home based management of fever. Barriers to task shifting included reluctance to change, protection of professional turf, professional boundaries and regulations, heavy workload and high disease burden, poor planning, lack of a task shifting champion, lack of guidelines, the name task shifting itself, and unemployed health professionals.

There were both positive and negative views on task shifting: the positive ones cast task shifting as one of the solutions to the dual problem of lack of skills and high demand for service, and as something that is already happening; while negative ones saw it as a quick fix intended for the poor, a threat to quality care and likely to compromise the health system.

**Conclusion:**

There were widespread examples of task in Uganda, and task shifting was mainly attributed to HRH shortages coupled with the high demand for healthcare services. There is need for clear policy and guidelines to regulate task shifting and protect those who undertake delegated tasks.

## Background

The human resources for health (HRH) crisis in the East, Central and Southern African (ECSA) region is attributed to a number of factors, including high disease burden, high demands due to HIV/AIDS, and low capacity for the production of health professionals in most of the ECSA countries [1]. Task shifting, the rational redistribution of tasks among health workforce teams, with specific tasks moved from highly qualified health workers to health workers with shorter training and fewer qualifications in order to make efficient use of the available human resources [2] has been proposed as one of the ways to mitigate the impact of health worker shortfalls; and as an opportunity for countries to build equitable and sustainable health systems [3-5].

The East, Central and Southern African Health Community (ECSA HC) is an inter-governmental organization that fosters regional collaboration in health. ECSA member states have pronounced themselves on task shifting on a number of occasions. For instance, during the 46^th ^Health Ministers Conference (February 2008), the ministers committed themselves under Resolution ECSA/HMC46/R4 which, inter alia:

### Urged member states to

*Develop and implement policies, guidelines and training curricula on task shifting among health care providers that will allow mid-level cadres to carry out specifically identified activities such as caesarean section, manual removal of the placenta, manual vacuum aspiration and insertion of norplant and interuterine devices; and in turn shift non/less technical duties from mid-level to lower-level cadre staff such as community based distributors of contraceptives and patient attendants by 2011; *and

### Directed the secretariat to

*Support countries to develop and implement policies and guidelines on task shifting among health care provides by 2010 *[6].

However, during a meeting of the ECSA College of Nursing (ECSACON) in Harare, Zimbabwe, in November 2008, the nursing fraternity in region expressed their anxiety over the possible introduction of task shifting, in recommendations whose preamble included the statement:

"*Disturbed by the confusion occasioned by moves to introduce or adopt task shifting approaches at country level;*....", with a plea for national consultations on the best way to adopt task shifting [7].

In response to those resolutions, recommendations and concerns, ECSA HC Secretariat, with support from USAID/HPI, undertook a case study on task shifting in Uganda, with the broad objective to establish what obtained on the ground, while the specific objectives of the study were to:

1. Understand the policy and programmatic implications of task shifting in relation to the roles, responsibilities and workload of health workers within the context of providing quality HIV and AIDS care and support services.

2. Understand the policy and programmatic implications of task shifting in the utilization of community based health workers and/or people living with HIV and AIDS (PLWHA) to provide peer counseling and related services

3. Assess the attitudes and perceptions of health workers regarding task shifting.

The choice of Uganda for the case study was partly informed by its long history of task shifting, dating as far back as 1918 when the Ugandan health service pioneered the use of a cadre of medical professionals originally referred to as licentiates, later medical assistants, and currently known as clinical officers [8]. Another long-standing example is in pharmaceutical services where due to the lack of pharmacists, pharmaceutical technicians (or dispensers) staff most of the hospitals and render the full range of pharmaceutical services.

A preliminary report of the study was presented at the 50^th ^ECSA Health Ministers Conference [9].

## Methods

### Study design

This was a cross sectional, qualitative study conducted between 6 August and 22 August 2009 through 34 in-depth key informant interviews and eight (8) focus group discussions (FGDs, with 80 participants), using pre-tested tools. We focused on the same major areas in the in-depth interviews and focus group discussions, i.e. (i) understanding task shifting, (ii) examples of task shifting, (iii) policy, guidelines and institutional arrangements for task shifting, (iv) political commitment to task shifting, (v) enabling factors and barriers to task shifting, (vi) opportunities for scale-up and HR capacity building through task shifting, and (vii) personal views on task shifting. The areas of inquiry were deliberately kept broad to enable the respondents to share experiences beyond HIV/AIDS service delivery, in line with Objective 3.

### Respondents

The key informants and focus group participants were selected by convenient sampling, with attempts made to be inclusive of various levels of the health system and different parts of the country. The health service in Uganda is delivered through a decentralized network at local, district, regional, and national levels. The lowest level of the system is the Health Centre I (HCI or village health committee) followed by Health Centre II (HCII), Health Centre III (HC III), and Health Centre IV (HC IV). The fifth level of care is the district or general hospital, which refers patients to the regional referral hospital (RRH), which may then refer patients to the national referral hospitals. The key informants included policy makers, HR managers, health workers, health worker leaders and managers, and student nurses. FGDs were held only with frontline health workers and students, and in all instances were based on participant availability.

### Setting

The respondents were from the Ministry of Health (MoH) headquarters, the Uganda Peoples Defence Forces (UPDF) Medical Services, Health Service Commission, Uganda Nursing and Midwives Council, Uganda Allied Health Professions Council, National Referral Hospital Mulago, National Referral Hospital, Butabika, one partner organization (UNFPA) in the Central Region; Masaka Regional Referral Hospital, Masaka School of Registered Comprehensive Nursing, Ibanda District Health Service in the South Western region; Jinja Regional Referral Hospital, Mbale Regional Referral Hospital, Budaka District Health Service, two health centres - one level IV and one level III - from Budaka District, a rural district, and one faith-based organization (FBO) in the Eastern region.

Though there were no specific documents on task shifting policy or guidelines, the National HIV/AIDS treatment guidelines and the Health Sector Strategic Plan II (HSSP II) were reviewed to complement information obtained from the interviews and FGDs.

### Data analysis

Notes from the interviews and FGDs were organized under major thematic areas, using content analysis and open coding. The major themes mirrored the focus areas identified in the study tools. A narrative approach is used to present the data.

### Ethical considerations

The protocol for the study was approved by the Institutional Review Board (IRB) of HPI Futures Group, as part of the Task Order I activities, and subsequently the study was cleared by MoH Uganda. At the commencement of the study, the Director General of Health Services provided letters introducing the study team to various stakeholders. Telephonic appointments were made ahead of the visit by the team. All participants were briefed on the study after which they signed an informed consent form. Except where respondents specifically indicated they could be quoted, the information is presented anonymously. The timing of the interviews or FGDs was such that there was no disruption in service delivery at any of the facilities, and where students were involved, the FGDs were held outside their normal learning activities, and with the full blessing of the school authorities. The respondents did not receive any compensation for their participation in the study.

### Limitations

The study covered only facilities along the East-West axis of the country; it was not possible to make contact with the relevant authorities in the northern region for inclusion in the study. Secondly, the private sector was not included in the case study due to lack of timely communication with the various role players, such as the FBOs and professional associations. However, with the interviews and FGDs that were conducted data saturation was reached, so it is likely that a more extensive study would not have provided additional information. We did not have access to the staffing profile for the public health sector in Uganda at the time of the study, and it is therefore not possible to comment on how different the convenient sample we used was from a possible random sample. Nevertheless, the qualitative approach used in the study makes the need for a random sample less crucial.

## Results

### Respondents

Thirty four (34) key informant interviews were conducted, with a cross section of policy makers, employers, health service managers and health workers. In addition, eight (8) focus group discussions were held in six (6) facilities (two each at Masaka Comprehensive School of Nursing and Jinja Regional Referral Hospital), and one each at Butabika National Referral Hospital, Masaka Regional Referral Hospital, Mbale Regional Referral Hospital, and Budaka Health Centre IV.

### Understanding task shifting

Most of the key informants had heard of task shifting. Policy makers and the more senior health professionals knew task shifting as defined by WHO, but lower level health workers had less accurate descriptions of task shifting. Misconceptions of task shifting included "dumping of tasks to others", "neglecting [one's] duties", and "making unauthorized delegation of duty". Once the misconceptions were corrected, the informants stated that task shifting was neither new nor uncommon in Uganda.

Task shifting was understood as occurring not only vertically, but also horizontally, for instance general surgeons undertaking procedures such as hysterectomy which should be done by the gynaecologists, dental surgeons doing the work of a general practitioner, midwives undertaking general nursing duties, nurses taking on dispensing duties, and doctors managing health facilities.

### Examples of task shifting in Uganda

The respondents cited numerous examples of task shifting, ranging from medical officers who undertook specialist surgical procedures, the management AIDS patients by various cadres, nurses and clinical officer doing the clinical duties supposed to be done by medical officers; and community health workers treating malaria cases.

The historical context of the Uganda health service included the development of a mid-level cadre "between the doctor and the nurse"; these have been variously known as licentiates, medical assistants and presently as clinical officers. The clinical officers take on a full range of clinical services at health centre levels III and IV, while in the hospitals they typically man the outpatients departments. However, clinical officers are no longer confined to outpatient care in the hospitals where there are doctors; a case in point was the Paediatrics Department at Mbale Regional Referral Hospital where clinical officers work alongside paediatricians and medical officers, following standard guidelines for fluid expansion under the Fluid Expansion as Supportive Therapy for Sick children (FEASTS) Project.

A frequently cited example was in pharmaceutical services where due to lack of pharmacists, the pharmaceutical service relies heavily on pharmaceutical technicians (dispensers) who staff most of the hospitals. At lower levels of the health system, pharmaceutical services are in the hands of nurses who do all the dispensing.

Nurses mentioned a number of areas where they were involved in task shifting -beyond the scope of practice of the traditional nurse - including putting up intravenous (IV) lines, and engaging in patient assessment, diagnosis and prescribing.

A common form of task shifting was the involvement of the "attendants" - patients' relatives or family members - who often take on roles such as feeding and bathing of in-patients, the collection of medicines from the pharmacy (and sometimes they purchase medicines outside the hospital in case of stock-outs), taking specimens to the laboratory and collection of laboratory results.

At village level, the National Malaria Control Programme introduced the cadre known as Community Medicine Distributor (CMD) under the home-based management of fever (HBMF). In HBMF, village volunteers were trained as community medicine distributors (CMDs) to recognize and treat uncomplicated fevers in children, and to recognize and refer severe/complicated malaria through features such as diarrhea and vomiting, failure to feed, convulsions or not passing urine. The treatment for malaria under HBMF was based on pre-packed medicines (Homapak) distributed in two age-specific colours, red for those between six months and two years, and green for children between two and five years. Children with fevers accessed treatment within 24 hours. A revised HBMF programme with current first-line regimen was due to be rolled out country-wide in September 2009.

With specific reference to HIV and AIDS, respondents readily cited examples of task shifting across all the four task shifting levels [2]. Clinical officers and nurses had reportedly taken on more clinical roles, guided by treatment guidelines from the National STI and AIDS Control Programme. There was also the involvement of lay service providers, such as counselors and treatment supporters, and people living with HIV and AIDS (PLWHA) in prevention, treatment, care and support of those infected and affected by HIV. It was widely acknowledged that management of HIV/AIDS services relied heavily on task shifting approaches; indeed, even respondents who seemed vehemently opposed to "task shifting" mellowed when presented with scenarios of PLWHAs as treatment supporters or as peer counselors.

Most respondents reported no objections to CHWs taking on roles such as nutritional support, prevention of HIV transmission (including PMTCT), HIV treatment literacy, counseling HIV positive women/men on family planning, provision of sexual and reproductive health information and referrals, community treatment access advocacy, stigma reduction and women empowerment, advising PLWHA on income generating projects and First Aid in emergencies. However, it was unanimously felt that core decisions such as initiation and variation of therapy and monitoring of progress should be left to appropriate health professionals.

A summary of specific examples of task shifting is presented hereunder:

Task Shifting I: Examples of delegation of diagnostic and prescribing privileges from doctors and specialists to non-physician clinicians such as nurses and clinical officers included:

• Tasks shifted to clinical officers, through deliberate and formal strategies such as in the guidelines for ART and PMTCT offered at HC III where the highest qualified clinician is the clinical officer; or circumstantially where there are no doctors. Where there are no clinical officers, the nurses may take on the clinical roles traditionally reserved for the doctors. The latter was informally done, with no written guidelines or procedures.

• Ophthalmic clinical officers render care such as cataract surgery, previously the preserve of ophthalmologists.

• Psychiatric clinical officers (PCO), a cadre in the mental health service, have helped expand mental health services in the country. The psychiatric clinical officers cover the same scope as the psychiatrists, but are more community-oriented than the psychiatrists who tend to be mainly hospital based

• The prescription of morphine has been delegated to clinical officers and registered nurses - the palliative care practitioners - who receive additional training from Hospice Africa. Previously morphine was reserved for inpatients under prescription by a medical officer.

Type II Task shifting: where nurses/midwives take on clinician roles previously reserved for medical officers or clinical officers:

• Nurses never used to insert IV lines, but presently it has become routine for them to do so in the hospitals, especially in upcountry facilities - there has been a gradual shift of the task to set up IV lines from doctors to nurses, but this is not protected by existing laws and regulations.

• Manual vacuum extraction, manual removal of the placenta and manual vacuum aspiration that were previous the domain of doctors only are now carried out by midwives with adequate training, supported by changes to the laws governing the scope of practice of the midwife and corresponding changes to the re-service curriculum.

• At the Infectious Diseases Institute (IDI) in Mulago Hospital, nurses were trained and given guidelines to manage some categories of AIDS patients that presented for review.

• Comprehensive enrolled nurses run health centre level II facilities, which involves all the diagnostic and prescribing duties, in addition to the nursing and midwifery ones.

• Many nurses/midwives received only midwifery or only nursing training, and yet when they were posted to the health facility they had to offer both types of services (horizontal task shifting).

Type III task shifting, where nurses and midwives tasks are delegated to nursing assistants or nursing aides or community health workers (CHWs):

• Home-based management of fever (HBMF) which relies on CHW as CMDs

• On many wards, in hospitals and health centres, due to nursing shortages, sometimes the nursing assistant may be the only staff on duty; occasionally there is one nurse and one nursing aide looking after over one hundred patients - the duties are then split between the two staff - and each carries out the same tasks.

• In the TB Control Programme, a cadre of staff that are trained in microscopy, the microscopists who do sputum smear microscopy (ZN stain and reading of slides), often help with other laboratory services due to lack of laboratory technologists.

Type IV task shifting, Task Shifting IV: Delegation of tasks from nurses/CHWs to expert patients or patients' relatives (attendants)

• Formal arrangements at the Burns Unit, Mulago Hospital where patients' relatives are trained to assist in the on-going care of their patients.

• CHWs and community members involved in delivery of expanded programme on immunisation (EPI) services.

• Management of information systems, referrals and medicines distribution which were sometimes often shifted to non-professionals due to shortage of skilled professionals.

• Delivery of HIV/AIDS services - counselling, treatment support and home care

• At IDI, registration and follow-up reminders were done by PLHWA.

• PLHWA and lay counsellors involved in care and support of HIV/AIDS patients.

### Policy, guidelines and institutional arrangements

At the time of the study there was as yet no written policy on task shifting, but the process was underway towards development of policy, with support from UNFPA. Information from the ministry was that the policy direction was towards task shifting that would "not compromise quality of care; and task shifting that allows some tasks to be shifted downwards or upwards, as the case may be." According to the Director of Health Services:

"Task shifting should not be task piling; and there should be no dumping of tasks. We need to ensure that task shifting happens in a team, based on which cadres are available, which tasks need to be undertaken and who has which competencies. It shall not be arbitrary or cast in stone."

Political commitment was reported at the highest level, with the Minister of Health very supportive of task shifting. The regulatory councils expressed eagerness to be involved early to ensure that professional standards and patient safety were maintained, and that all shifted tasks were regulated at appropriate levels of competency.

There were guidelines through which task shifting was happening in some specific porgrammes, as in the HBMF programme, the situation at IDI, the management of HIV/AIDS nationwide and the involvement of patient relatives in the care of burns patients at Mulago, as already cited.

Whereas there were no legal instruments, written policy or guidelines to support sector-wide task shifting, existing practice was permissive of task shifting within the facilities, with no strict enforcement of "scope of practice" rules. The feeling among front-line health workers was that task shifting may not require new laws, but rather explicit policy and guidelines for operational level implementation and monitoring. However, respondents from regulatory bodies felt changes would be required to some laws to expand the scope of practice for certain cadres, for example if clinical officers were to do Caesarean Sections.

The UPDF Medical Services, which are independent of the MoH, had undertaken a process to develop a special cadre, the Company Medic to ease the severe shortage of skills. The steps taken included obtaining buy-in at the highest level with endorsement from the Army Commander, followed by development of policy and guidelines, engagement of health education and education curriculum experts to develop the course for the Company Medic. The new cadre was expected to be equipped with the basic knowledge and skills to manage conditions common in the military situation. At the time of the study plans had been finalized for the first batch of company medic recruits to begin their training at Jinja Regional Referral Hospital.

Some respondents indicated the need for caution in the development of task shifting policy or guidelines, for any move to make it widespread would be met by opposition from the professionals and the public alike. Those of this view preferred task shifting to be confined to specific issues, in special circumstances, for instance clinical officers trained to render care for AIDS patients had not met opposition from other professionals or the public.

### Facilitating factors for and barriers to task shifting

Many explanations were given and many suggestions made on what was enabling or would enable task shifting to happen in Uganda; and on what barriers there were to the implementation or development of task shifting in Uganda. Remarkably, the reasons in support of the suggestions were similar across a wide range of respondents. The main suggestions are summarized in Table 1 below.

**Table 1:** Facilitating Factors and Barriers to Task Shifting in Uganda

Facilitating factors	Barriers
• Policy on task shifting	• The name task shifting
• Standard Operating procedures	• Poor health worker pay and conditions of service
• Better candidates entering the professions	• Lack of awareness
• Evidence of successful task shifting	• Lack of legal protection
• Lax regulatory environment	• Lack of policy and guidelines
• Poor law enforcement	• Corruption
• Institutional or programmatic guidelines	• Poor planning, unregulated task shifting
• High demand for health services	• Professional boundaries and regulation
• Scarcity of skills	• Poor community attitude
• Focussed initiatives, e.g. home based management of fever	• Professional protectionism
• International commitments, e.g. MDGs	• Heavy workload and high disease burden
• Functioning referral chain	• Reluctance to change
• Greater awareness on what task shifting was all about	• Limited knowledge and skills
• A task shifting champion	• Unemployment or lack of job opportunities for health professionals

### For scaling up and HR capacity building

Views on what opportunities task shifting presented for scale up and capacity building seemed to be influenced by the history of a freeze in employment in the public service in the late 1980s to mid-1990s, and by the subsequent decentralization of health service administration to districts which are now responsible for recruitment of health professionals. A consequence of those two developments was that some health professionals were not absorbed into the health system. According to a key informant responsible for the health workforce:

*"That is an anomaly Uganda cannot afford. As long as we need the professionals, and they are within the country, we should employ them. The only way that will happen is to recentralize the recruitment and deployment of health workers so that the Health Service Commission can keep track of all those within the public sector*."

The presence (or perception) of unemployed professionals made the option of task shifting difficult to implement, according to one Assistant Commissioner of Health Services, because whatever was suggested appeared like going for the cheaper alternative, which may then limit knowledge transfer and capacity building. Indeed, for many respondents, given the hypothetical situation where all professionals were employed, there would be no problem in building the capacity of other cadres to take on some of the tasks, under supervision.

Those who were out of necessity taking on tasks not originally within their scope of practice (such as nurses setting up IV lines) recommended that such skills be introduced during in-service training, even without a change in laws since in reality they were executing such tasks. As one senior nurse stated:

"During my training I was never taught how to put up a drip, and for my first twenty years as a nurse all I did was prepare the trolley for the doctor. When I got to this hospital, even the nursing assistants were laughing at me for not knowing how to put up a drip. That is demoralizing."

Where task shifting initiatives was introduced formally, there had been capacity building through training. An example was within the reproductive health domain where midwives and clinical officers received skills training (pre-service and in-service) for post-abortion care, manual vacuum aspiration and long term methods of family planning such as insertion of Norplant.

There were cited a number of constraints to HRH capacity building within the task shifting context, including poor remuneration and working conditions which made it difficult to retain skilled professionals who would impart skills to others, and yet some existing posts were frozen due to expenditure ceilings, with perceived reluctance of the National Treasury to open up more posts in the health sector.

An approach suggested in one of the focus group discussions was long-term planning taking into account the full needs of the health system, based on a thorough skills audit and attention to health worker concerns such as low pay, poor state of existing facilities and lack of professional development prospects. In agreeing with those sentiments, a senior manager of a regional referral hospital stated:

"If government employed more skilled professionals, for instance doctors, it would be possible to enforce 8-hour shifts for doctors round-the-clock, and the need for task shifting during the night would not arise. At present, the doctor is expected to work 24 hours, every day, for no additional compensation, and so does not show up in the night."

The approach used in the mental health service illustrates effective capacity building through task shifting. As a result of the growth of number of PCOs, the specialized mental services have been extended beyond the national referral hospital to regional referral hospitals which now have specialized psychiatric units, and from regional hospitals to the district hospitals where the PCOs are in charge of psychiatric services. The psychiatric nurses are also equipped with more skills and are able to recognize, initiate treatment or refer psychiatric patients. That had contributed to the scale up of mental health services in the face of very few psychiatrists.

Opportunities for scaling up best practices in task shifting, building on existing approaches, included efforts by the Malaria Control Programme to roll out the new HBMF, using a new regimen, based on the earlier experience with the CMDs and the success of the pilot studies within a number of districts. The management of HIV and AIDS through tasks delegated to various cadres also presented opportunities for scaling up of services, and as illustrated by the example at IDI, it was through such strategies that the Institute had been able to expand its services.

The situation at IDI, however, also illustrates a problem with successful task shifting. The institute has become so efficient at what it does that at present the clinical service is overwhelmed by the numbers. One of the clinical managers remarked:

*"We created the demand for our services, and now clients are abandoning other providers and coming to us. We simply cannot cope with all who want to be under our care. As much as it breaks my heart every time I have to turn people away, I have to do it on a daily basis"*.

The opportunity for scaling up any initiative must therefore be tempered with caution regarding its sustainability and capacity to cope with expected increases in numbers seeking the services. The balancing act would involve the choice of scaling up the initiatives and the possibility of overloading the system, or to move cautiously within the resource constraints and risk not reaching many of those in need.

### Selected respondent views on task shifting

The interactions with the respondents included as one of the last questions, "So what would you say about task shifting?" This attracted many views on task shifting, which were broadly positive or negative. Those in favour of task shifting tended to describe it in positive terms, incorporating elements such as "delegation", "where necessary", or "appropriate"; while those against it saw it in terms such as "task dumping", "cheap", "sub-standard care", and "compromised quality of care". Some of these are presented verbatim in Table 2.

**Table 2:** Selected Respondents' Views on Task shifting

(a) Positive Views	(b) Negative Views
*• Task shifting is real and it is safe when well managed. Even the doctors have come to accept that clinical officers can do many tasks. Let the regulations change to allow easier task shifting with protection of the health professionals *(Clinical officer*)*.	*• Not anybody can be a nurse, we need to look holistically at patient care and the best interests of the patient. Lawyers never let anyone but lawyers take on cases in court, why should we let nursing duties be handled by anyone else? *(Assistant commissioner, health services)
*• Task shifting works when well planned, with guidelines. Resistance is based on the need to protect professional turf, doctors feel undermined if clinical officers carry out surgical procedures *(Respondent from a regulatory council).	*• We know task shifting is a reality, but we don't have to shout about it as if it's the right thing. We must build capacity for the right people to render the necessary services. The quality of care always suffers when the less qualified take on higher tasks (*senior consultant doctor).
*• Task shifting is a very good approach; and for psychiatry it is the only solution. It would take very many years to train psychiatrists for the whole country - at present Uganda has only 26 psychiatrists for 30 million people*. (Mental health professional*)*	*• Task shifting has been adopted by rural facilities as a necessity; but without proper guidelines, it is a ticking time bomb *(principal nursing officer).
*• Task shifting should not worry any professionals, it is happening every day, and now we have a chance to do it properly. Even if clinical officers are allowed to do more surgery than they are doing now, it will not make the doctors any less important *(MoH official)	*• Considering the issue of circumcision, what happens when all the people have been circumcised? Such a waste of skills! *(Hospital manager).
*• Task shifting is happening, but should be followed by protection of the nurses when they go beyond their scope through documentation; and when they delegate to lower cadres, there should be proper supervision. (*Assistant commissioner, health services*)*	*• Task shifting can only be justified when a new structure of the health service is in place, the remuneration of the health workers is much better, and we still see gaps. Otherwise, we are undermining the health system by going for short sighted solutions such as task shifting *(senior consultant doctor).
*• Resistance to task shifting is all about protecting one's professional turf, that's all there is to it; no one is asking for task shifting to be implemented in a vacuum. So there is need for a change in mindset *(FGD participant).	*• Government should just get the right people for the right jobs. We have the trained people in Uganda, but we are operating with archaic structures that do not take into account the growth in population and the heavy disease burden *(Assistant commissioner, health services).
*• Task shifting can work, but should be introduced carefully, with clearly identified tasks to be shifted to specific cadres, under specific conditions *(Commissioner, health services).	*• It would be unwise to shift caesarean sections to clinical officers or midwives in Uganda; it is not that it should be only for doctors, but the level of clinical skills that are required to make the decision to operate should not be taken lightly. It would jeopardize patient safety if everyone were allowed to do caesarean sections *(senior consultant doctor).
*• Task shifting is a good thing that can work with proper regulation and legal protection. The framework already exists, but there is no supporting policy and implementing law as yet *(Commissioner, health services).	*• Task shifting is symptomatic treatment; a mechanism for the poor who are condemned to receiving treatment from sub-standard cadres; the rich are never for task shifted services. Success stories of task shifting in Mozambique and Malawi are exaggerated *(Commissioner, health services).
*• Task shifting is a good concept but there is no one pushing for it. We all appreciate it because of the shortage of skilled health workers, but it has to be done properly*. (senior medical officer)	

## Discussion

Task shifting is a reality in Uganda, spurning the whole range of task shifting options from type I to type IV [2,10]. The present study confirmed the long history of task shifting approaches, as documented by others [8,11,12]. However, at the time of the study there was no over-arching policy, and what guidelines existed tended to be programme-specific. Thus task shifting was happening often without legal protection for those who took on delegated tasks within the existing regulatory framework, save for institutional arrangements that permitted such task shifting. The need for proper policy and guidelines was emphasized by many respondents throughout the study.

The recent interest in task shifting strategies is largely attributed to the need to respond to the demand for HIV/AIDS services, especially in resource constrained settings (e.g. [3-5]], hence the focus of the present study on HIV/AIDS service delivery but in the context of the broader health care services. Uganda participated in the background studies that informed the Global Recommendations and Guidelines [2,10], and yet there were misconceptions on the concept of task shifting. At the time of the study the WHO Guidelines had not been disseminated, and even District Health officers were not up-to-date with the task shifting developments. That gap in information will have to be addressed for instance by briefing the DHOs who would in turn sensitise those who work within the districts.

Success stories of task shifting approaches in Uganda, such as those in reproductive health, HIV/AIDS care and mental health services, were associated with proper planning of programmes with operational guidelines to ensure protection of those taking on delegated tasks through supervision. Those successes, however, remained understated with little publicity. The work and role of the PCO, for example, was hardly known beyond hospitals with psychiatric units; the use of patient attendants at the Mulago Burns Unit was not common knowledge even among MoH officials; and health professionals in lower level health units appeared unaware of changes that empowered midwives to carry out procedures in post-abortion care. For scale-up of the task shifting initiatives, it will be helpful to document, disseminate and publicise such successes.

The name task shifting was unacceptable to some respondents; some praised initiatives such as home-based management of fever by CMDs, but when that was pointed out as an example of task shifting, they felt disappointed. There was a perception among some health workers that task shifting was being imposed on Uganda by WHO, which suggests that the concept had not been well presented to stakeholders such as front-line health workers. Indeed, there have attempts at international gatherings to move away from the term "task shifting" to "task sharing", for instance at the ECSACON Conference in August 2010 [13].

As indicated in the findings, there was political support and commitment to task shifting at the highest level, and the technical team within MoH was convinced of the rationale for task shifting, both of which will facilitate the development of policy and guidelines. That vantage point needs to be complemented by dissemination of the concept to the wider health sector to ensure buy-in and to disabuse health professionals of misconceptions that may breed resistance to the initiative.

The issue of recruitment and deployment of unemployed health professionals was cited as a barrier to scaling up task shifting initiatives. A suggestion was made that a skills audit and restructuring of the health system be done to establish the gaps and ensure that qualified professionals get first priority. Unless that is addressed, suspicions will remain among health workers that task shifting is a substitute for hiring professionals; especially with the perception that employers at the district level would rather employ less qualified candidates to save on the wage bill.

That the HRH crisis in Uganda makes task shifting inevitable vindicates views from elsewhere, for instance Mozambique [14-16], Tanzania [17-19], Ethiopia [19-22] and Malawi [23-25]. Nevertheless, there were many concerns from the health professionals regarding their protection should anything go wrong and fear for too much workload. In this respect, Uganda is not alone; similar concerns have been expressed elsewhere, with the caution that task shifting cannot be expected to make up for all the systemic short comings [e.g. [26-28]]. Be that as it may, services such as HIV/AIDS care and support, anaesthetic services and psychiatric services rely heavily upon task shifting approaches [11,12,29,30].

Bold and imaginative leadership will be required to steer the policy development and implementation processes and to manage any fall-out. For instance, should it be proposed that clinical officers or midwives do caesarean section, which seemed to be the least popular example of task shifting, the campaign would need such leadership to oversee the preparation of the case for that task shifting. It would also require the engagement of stakeholders such as obstetricians, doctors, and the communities. The other danger, as expressed in some FGDs, is that if task shifting were not done carefully, and in the absence of effective leadership, some professionals may abandon their work to others, and go for private practice on government time.

## Conclusion

Task shifting was happening in Uganda on a wide scale, at various levels of care, in many forms. The main driver for task shifting appeared to be low HRH density coupled with the high demand for healthcare services. Most of the task shifting was happening without enabling policy, regulations or legal protection for those who undertook delegated tasks, though there were successful examples of task shifting backed by institutional frameworks. The policy environment was supportive of task shifting, and the process was underway to develop a policy and guidelines for task shifting in Uganda. Not much publicity had been given to the concept of task shifting, and many health workers had misconceptions of what it was all about, in spite of successful examples of task shifting in the country. The future of task shifting in Uganda will depend on the strategy adopted and the extent to which concerns of health professionals are addressed.

## Abbreviations

ART: Anti-retroviral therapy/treatment; CHW: Community health worker; CMD: Community medicine distributor; DHO: District Health officer; ECSA HC: East: Central and Southern African Health Community; ECSA: East: Central and Southern Africa; ECSACON: ECSA College of Nursing; EPI: Expanded Programme on Immunisation; FBO: Faith Based Organisation; FEASTS: Fluid Expansion as Supportive Therapy for Sick Children; FGD: Focus Group Discussion; HBMF: Home-based management of fever; HC: Health Centre; HIV/AIDS: Human immunodeficiency virus/Acquired immune deficiency syndrome; HMC: Health Ministers' Conference; HPI: Health Policy Initiative; HR: Human Resource(s); HRH: Human Resources for Health; HSSP II: Health Sector Strategic Plan II; IDI: Infectious Diseases Institute; IRB: Institutional Review Board; IV (line): Intra-venous; MoH: Ministry of Health; PCO: Psychiatric clinical officer; PLWHA: People living with HIV/AIDS; PMTCT: Prevention of mother-to-child transmission; RRH: Regional Referral Hospital; STI: Sexually transmitted infection; UNFPA: United Nations Population Fund; UPDF: Uganda Peoples Defence Forces; USAID: United States Agency for International Development; WHO: World Health Organization; ZN (stain): Ziel-Neelsen (stain)

## Competing interests

The authors declare that they have no competing interests.

## Authors' contributions

YMD contributed to the study concept and design, developed the tools, conducted most of the interviews, facilitated most of the FGDs, coordinated the rest of the study in Uganda, analysed the data and drafted the manuscript and revised it to its final form with inputs from the co-author. SM developed the study concept, contributed to the finalization of the study tools, coordinated the approval process for the study with MoH Uganda, reviewed the results from the study and contributed to the drafting, revision and finalization of the manuscript. Both authors read and approved the final manuscript.

## Authors' information

YMD (MB ChB, PhD) is Senior Professor in the Department of Pharmacy at the University of Limpopo, South Africa, and a member of the Regional Network for Equity in Health in East and Southern Africa (EQUINET) Steering Committee. SM (BCur, MPH) is Manager, Human Resources for Health and Capacity Development (HRH & CB) at the ECSA Health Community, Arusha, Tanzania.

## Pre-publication history

The pre-publication history for this paper can be accessed here:

http://www.biomedcentral.com/1472-6963/12/61/prepub
